# Locally acquired typhoid fever outbreak linked to chronic carriage in Ottawa, Canada, 2018–2022

**DOI:** 10.14745/ccdr.v50i11a05

**Published:** 2024-11-07

**Authors:** Janice Zhang, Ann Jolly, Tram Nguyen, Monir Taha, Christina Lee, Antoine Corbeil, Esther Dapaah, Jeff Walker, Curtis Cooper, Jacqueline Willmore

**Affiliations:** 1 Canadian Field Epidemiology Program, Public Health Agency of Canada, Ottawa, ON; 2 Ottawa Public Health, Ottawa, ON; 3 Public Health Ontario, Toronto, ON; 4 The Ottawa Hospital, Ottawa, ON

**Keywords:** typhoid fever, *Salmonella* Typhi, epidemiology, public health, disease outbreaks, whole-genome sequencing, Canada

## Abstract

**Background:**

In Canada, *Salmonella enterica* serovar Typhi infections are uncommon and typically travel-related. In November 2021, Ottawa Public Health identified a link between two typhoid fever cases, with no recent history of international travel, to the same grocery store ready-to-eat counter.

**Objective:**

This report describes the outbreak response to a rare occurrence of chronic *S.* Typhi carriage in Ottawa, Ontario, Canada and provides recommendations for investigations of small-scale protracted outbreaks.

**Methods:**

We administered exposure questionnaires using a single interviewer approach, tested stool samples of contacts and food handlers, inspected food premises, collected food samples and reviewed takeout receipts. Social network, spatial and whole genome sequencing analyses were used to investigate additional possible links between cases.

**Results:**

Seven people with typhoid fever and onset from October 2018 to May 2022 were linked to an asymptomatic chronic *S.* Typhi carrier. Whole-genome sequencing confirmed that all eight isolates matched the outbreak cluster. All cases and carrier resided within an eight km radius in Ottawa. The chronic carrier worked as a food handler at various locations of a grocery store chain, including the implicated ready-to-eat counter. Transmission occurred via food handling, shared workspaces and social and household networks.

**Conclusion:**

The chronic carrier was excluded from food handling until successful completion of treatment and clearance testing. We overcame the challenges of a small but prolonged outbreak by identifying an asymptomatic carrier using a multi-method approach including whole genome sequencing and social network analysis.

## Introduction

Typhoid fever is caused by direct or indirect fecal-oral exposure from an individual infected with *Salmonella enterica* serovar Typhi (*S*. Typhi). The primary incubation period is typically eight to 14 days but can range from three to more than 60 days. Symptoms can be non-specific, ranging from fever and mild illness to severe and potentially fatal illness requiring hospitalization ( ((1))). While most infections resolve with treatment, approximately 2%–5% of those infected become chronic carriers who can continue to transmit the bacteria for many years, often without symptoms ( ((2))).

Typhoid fever is a notifiable disease in Canada, and in Ontario, cases are reportable to the local Medical Officer of Health ( ((3,4))). Due to historic improvements to drinking water, sanitation and food safety, typhoid fever is uncommon in Canada ( ((1,5,6))). Between 2012 and 2021, an average of 140 cases per year were reported (average rate 0.4 per 100,000 population) ( ((6))). Most infections are associated with recent international travel, particularly visiting of friends and relatives in endemic regions ( ((7–11))). The most recent published report of a locally acquired typhoid fever outbreak in Canada was in Ontario in 1990, linked to consuming raw contaminated imported shellfish ( ((12))).

Between 2016 and 2020, an average of four cases of typhoid fever were reported per year in Ottawa, Ontario. In November 2021, Ottawa Public Health identified two locally acquired typhoid fever cases (cases D and E), residing 2.6 km apart, who had been reported two months apart, in September and November 2021, respectively. Case interviews identified linkage to a grocery store ready-to-eat food counter, one as a customer (case D) and the other as an employee (case E). An outbreak was declared in November 2021 and a multi-disciplinary team was assembled to identify the source of the outbreak and implement control measures. Preliminary whole-genome sequencing (WGS) confirmed these cases were related.

This report describes the outbreak response to a rare occurrence of chronic *S.* Typhi carriage in Canada and highlights the strengths of using a multi-method approach to overcome the challenges of investigating small-scale protracted outbreaks.

## Methods

### Case definition

A confirmed outbreak case was defined as a resident or visitor to Ottawa with laboratory confirmed *S.* Typhi infection and an isolate matching the outbreak cluster within 10 alleles by whole-genome multi-locus sequence typing (wgMLST). Probable cases included persons with laboratory confirmed *S.* Typhi infection awaiting WGS and an epidemiological link to a confirmed case. Suspect cases included persons with laboratory confirmed *Salmonella* infection awaiting serotyping and WGS, who had clinically compatible signs and symptoms of typhoid fever and an epidemiological link to a confirmed case. The case definitions do not specify a time frame as the potential period of exposure was unclear.

Close contacts were defined as household members, colleagues working in proximity and sexual partners of confirmed cases. Testing for *S.* Typhi was offered to all close contacts, with or without symptoms, using two stool samples collected at least 48 hours apart.

### Epidemiological investigation

Ottawa Public Health reviewed all typhoid fever cases reported in Ottawa since 2017 to identify any other potentially non-travel related cases. As much as possible, cases were interviewed by a single interviewer and some interviews were conducted in person. Cases were first interviewed using the Ontario standardized Salmonellosis Investigation Tool which gathers exposure information, including a detailed food history, for the seven days prior to symptom onset. Once *S.* Typhi infection was suspected or confirmed, cases were re-interviewed using the Ontario Typhoid Fever Investigation Tool which focuses on exposures in the three to 60 days before symptom onset ( ((13))). The interview included questions about occupation, personal and close contacts’ travel and consumption of high-risk foods. Additional questions were added on social contacts, country of origin, date of arrival in Canada and specific exposures based on evolving hypotheses. Cases reported prior to November 2021 were re-interviewed with these additional questions.

Social network and spatial analyses were used to generate hypotheses regarding missing epidemiological links. Addresses of cases and common exposure locations were mapped using the geoOttawa application and statistically significant geographic clustering was confirmed using SaTScan version 10.0. Social networks comprised cases and contacts as nodes with links being sharing meals from common sources, preparing food eaten by cases or contacts and working or socializing together, indicating shared bathroom use. As many clients forget contacts ( ((14))), we supplemented interviews by reviewing takeout receipts from digital food delivery apps and public social media accounts to further identify common exposures. Social network diagrams were created using Pajek version 5.14.

### Laboratory investigation

In Ontario, all *S*. Typhi isolates undergo WGS at Public Health Ontario through the PulseNet Canada program ( ((15))). The programmatic criterion to initially identify an *S.* Typhi genomic cluster is two or more isolates (including at least one clinical isolate), identified within 60 days, with 10 or less allele differences by wgMLST. Given the lack of international travel reported by the two index cases, a broader investigation of all *S.* Typhi isolates in Canada identified by PulseNet Canada since 2017 (when WGS was implemented for all *Salmonella* isolates) with 25 or less allele differences by wgMLST was performed to identify any potentially related cases ( ((15))). In Ontario, antimicrobial susceptibility testing is routinely performed on *S.* Typhi isolates.

### Environmental investigation

Public health inspectors from Ottawa Public Health inspected the grocery store ready-to-eat counter and collected food samples. They observed food handling practices, provided education on hand hygiene, compiled names of food handlers and inquired about illness and travel history. An anonymous survey was also distributed to food handlers to gather information on gastrointestinal and biliary tract illness history. Food handlers were tested for *S.* Typhi using three stool samples each collected at least 48 hours apart, regardless of symptoms.

## Results

**Figure 1:**
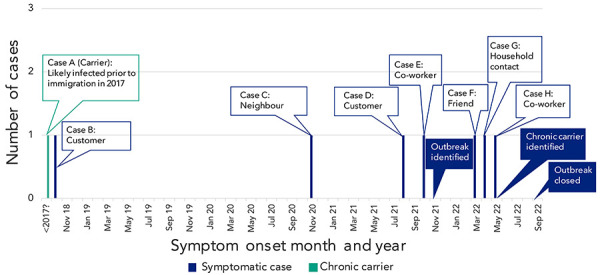
Epidemiological curve of *Salmonella enterica* serovar Typhi confirmed outbreak cases by symptom onset month, Ottawa, 2018–2022

Seven confirmed cases of typhoid fever with illness onset between October 2018 and May 2022 were linked to an asymptomatic carrier (**Figure 1**). All seven symptomatic cases were interviewed twice. The chronic carrier was interviewed first as a contact and then as a case. Twenty-eight close contacts were also interviewed but no additional cases were identified. Among the seven symptomatic cases, common symptoms included fever (n=7 cases), malaise (n=6), diarrhea (n=5), abdominal pain (n=5) and headache (n=4). Six cases were hospitalized for a median of nine days (range: 5–22 days) and one case received treatment in the emergency department; all recovered. All seven symptomatic cases and carrier resided within an eight km radius in Ottawa. Cases and carrier had a median age of 28 years old (range: 8–50 years) and six (75%) were male. Five of eight (63%) were immigrants to Canada (year of arrival ranged from 1997 to 2019), including four from the same country of origin, but no cases reported recent international travel.

Following the identification of the two index typhoid fever cases (cases D and E) in November 2021, laboratory investigations identified two additional cases (cases B and C) clustering by WGS with illness onset of October 2018 and November 2020, respectively (Figure 1). Of these four initial cases, three (cases B, D, and E) had an epidemiological link to the same grocery store ready-to-eat-counter as either customers (cases B and D) or an employee (case E). At that time, an exposure could not be established for case C. Given the long intervals between case illnesses and epidemiological linkage with the ready-to-eat counter, it was hypothesized that a likely source of transmission was a food handler with chronic *S.* Typhi carriage. Therefore, the initial outbreak investigation focused on the ready-to-eat counter.

Food premises inspections did not identify a source of transmission and no critical infractions were observed. Three food handlers were identified as working at the ready-to-eat counter during the incubation period for cases D and E but were not employed when case B was ill. All three food handlers submitted stool samples which were negative and none reported recent gastrointestinal or biliary tract illness. *Salmonella enterica* serovar Typhi was not detected in any food samples taken from the ready-to-eat counter.

A fifth case (case F) was reported in March 2022. This person had no known epidemiological linkage to the grocery store. They had a common country of origin with case E, suggesting a possible alternative social linkage. The person was interviewed to elicit possible social contacts working as a food handler or connected to previously reported cases; however, they were reluctant to provide information on their contacts.

The sixth (case G) and seventh (case H) cases were reported in May 2022. Contact tracing identified case G as a close contact of case F as well as sharing a common country of origin with cases E and F. Although case G may have been infected via close contact with case F, a common social network between cases F, G and an unidentified chronic *S.* Typhi carrier was also hypothesized. Case H was employed at another location of the same grocery store chain as a janitor. They were not a food handler and did not consume food from the ready-to-eat counter.

Further interviewing identified a short-term household contact of case G who worked as a food handler for the implicated grocery store chain since 2017 at various locations, primarily working at the ready-to-eat counter. This food handler (case A) also shared a common country of origin with cases E, F and G. We hypothesized that this newly identified food handler had chronic *S.* Typhi carriage acquired prior to immigration to Canada in 2017 from their country of origin (Western Pacific region). They were contacted to gather information and request stool testing. Stool cultures came back positive for *S.* Typhi. They were asymptomatic and reported no prior history of gastrointestinal or biliary tract illness.

**Figure 2:**
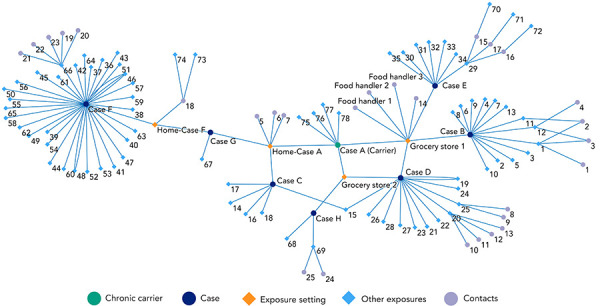
Social network diagram^a^ showing linkages between *Salmonella enterica* serovar Typhi outbreak cases and the chronic carrier, Ottawa, 2018–2022 ^a^ Social network diagram generated using Pajek version 5.14

**Figure 3:**
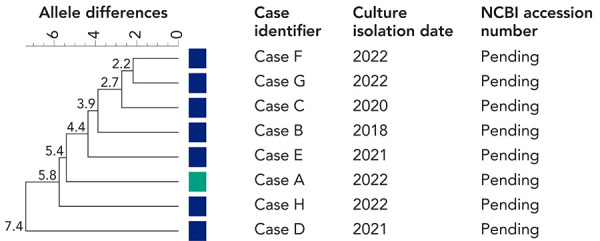
Phylogenetic tree of *Salmonella enterica* serovar Typhi outbreak-associated isolates, Ottawa, 2018–2022^a^ Abbreviation: NCBI, National Centre for Biotechnology Information ^a^ Whole-genome multi-locus sequence typing (wgMLST) dendogram generated using BioNumerics version 7. Based on 4,148 *Salmonella* Typhi alleles. The wgMLST allele differences indicated at the nodes were calculated using unweighted pair group method with arithmetic mean (UPGMA)

Epidemiological evidence strongly suggested that case A (carrier) was the source case for the outbreak as there were epidemiological links to all cases. Cases B, D, E and H were linked via two grocery store chain locations. Social network and spatial analyses established an epidemiological link with case C as neighbours in the same multi-unit complex (however, with no known direct contact) and with cases F and G as social or household contacts (**Figure 2**). Further, laboratory evidence revealed that all eight outbreak-related *S.* Typhi isolates were closely related within seven alleles by wgMLST (**Figure 3**), and there were no additional *S.* Typhi isolates matching this cluster in Canada since 2017 within the range of 0–25 alleles.

### Public health response

Individuals who met the confirmed, probable or suspect case definitions were excluded from working as food handlers, childcare workers or healthcare providers, until completion of antibiotic treatment and submission of three negative stool samples each collected at least 48 hours apart. Cases and close contacts were counselled on transmission, personal and hand hygiene, food handling, risk of *S.* Typhi carriage and safe sexual practices.

In May 2022, once local transmission was confirmed, an alert to healthcare providers in the region was issued about typhoid fever risk in patients without a travel history.

Case A (carrier) was excluded from food handling work in May 2022. They were referred to an infectious disease specialist and underwent successful susceptibility-informed antibiotic treatment. After treatment, they were cleared to return to work following there consecutive negative stool samples collected at least 48 hours apart ( ((16))). In total, case A (carrier) was excluded from food handling work for 59 days and had to find alternate employment due to financial hardship. The outbreak was declared over in September 2022, 120 days (two potential incubation periods) after the onset date of the last outbreak associated case. As of July 2024, no new cases have been identified matching the outbreak cluster.

## Discussion

This is the first reported outbreak of locally transmitted typhoid fever in Canada since 1990. Seven cases of typhoid fever over four years (from 2018 to 2022) were linked to an asymptomatic chronic *S.* Typhi carrier who worked irregularly as a food handler at various locations of a grocery store chain. Transmission occurred through food handling, shared workspaces and social and household networks. Although the outbreak was relatively small, six of seven cases were hospitalized with significant morbidity. This investigation highlights some of the challenges of identifying and managing a typhoid fever outbreak as well as the strengths of using multiple epidemiological, laboratory and environmental investigation methods during outbreak responses.

Characteristics of this outbreak are similar to others reported from non-endemic, high-income countries ( ((5))). Locally acquired typhoid fever outbreaks reported in the United States since the 1960s had limited secondary transmission and were often associated with a primary case of chronic *S.* Typhi carriage involved in food handling ( ((5,17–19))). This outbreak shares some of the challenges noted in these previous investigations. First, outbreaks caused by chronic *S.* Typhi carriage may be difficult to detect as carriers can shed bacteria intermittently for many years, potentially causing infections over a long period of time ( ((17))). Second, a small number of cases and long incubation period can make it more challenging to generate source hypotheses. Finally, given that typhoid fever cases have become infrequent in non-endemic countries, public health representatives may have limited experience managing such outbreaks ( ((18))).

This outbreak was difficult to identify due to the span of multiple years between cases. While the first clinical case was reported in 2018, the outbreak remained undetected until local public health nurses noted a common exposure between the two cases reported in 2021. Although WGS is routinely performed for *Salmonella* isolates in Ontario, the genomic linkage between cases was not flagged by the laboratory at the time due to the 60-day limit for initial PulseNet Canada cluster assignments. This illustrates the need to monitor and investigate any typhoid fever case for potential spatiotemporal and epidemiological linkages and to involve laboratory partners in surveillance and outbreak investigations to expand investigational options where relevant. It also highlights the potential benefit of expanding the PulseNet Canada relatedness analysis window beyond 60 days for *S.* Typhi, as recommended in another study examining typhoid fever outbreaks in the United States from 1999 to 2010 ( ((17))).

During this outbreak investigation, multiple factors limited the information available to generate source hypotheses, including the protracted length of time between cases, the varying modes of infection acquisition and the small number of cases. A case-control approach would have been problematic as responses to questionnaires are greatly affected by recall bias. Once we employed a multi-prong approach including WGS, social network analysis, a single interviewer and asymptomatic contact screening, we were successful in tracing cases to the primary source. Other outbreak reports have also highlighted the importance of using multiple methods in typhoid fever investigation ( ((19,20))).

The precarious nature of food handling work also hindered the investigation. The initial public health inspection of the implicated ready-to-eat counter failed to identify the carrier as an employee due to employment across multiple locations of the grocery store chain. In typhoid fever investigations, given the potential long period of exposure and transient food handler workforce, we recommend taking an extensive employment history of past, present and temporary food handlers. The exclusion from work for typhoid fever treatment and clearance also caused financial hardship to the food handlers. The negative impacts of excluding infected persons from work duties is likely to be shared within social networks, thus discouraging further cases and contacts from being interviewed and tested. Recent full compensation for those on medically mandated leave, such as that made available due to COVID-19 illness, presents a potential mechanism to facilitate employment insurance for other notifiable infections requiring exclusion from work ( ((21))).

Although there was no known direct contact, shared bathrooms or shared meals between case A (carrier) and case C (neighbour), we hypothesize that transmission potentially occurred through fomite contamination of common surfaces, such as doorknobs, railings or elevators. Likewise, although case H (the janitor) did not have known direct contact with case A or eat meals at the ready-to-eat counter, we hypothesize that acquisition likely occurred via common surfaces used by case A (carrier) at the grocery store (e.g., bathrooms). Training on hand hygiene and provision of proper personal protective equipment for janitors is essential to decrease the risk of enteric disease acquisition, as outside of healthcare and laboratory settings, janitorial work should not constitute an occupational hazard for infectious diseases ( ((22))).

Although outbreaks of typhoid fever are rare in Canada, they remain a risk particularly with international travel and immigration from regions where typhoid fever remains endemic ( ((20,23))). In addition, the emergence of drug-resistant *S.* Typhi in South Asia, increasingly observed in cases diagnosed in Ontario, has made effective treatment more challenging and prevention more urgent ( ((8,24))). The United States has reported cases of drug-resistant *S.* Typhi among individuals with no history of recent international travel ((25)). Surveillance and thorough case follow-up are essential to detect and control future outbreaks of typhoid fever ( ((19,24))).

## Conclusion

This outbreak report describes a rare outbreak of typhoid fever associated with chronic *S.* Typhi carriage in Canada and contributes to the literature to inform future investigations. An interdisciplinary investigation was key to discovering the transmission source. This outbreak demonstrates the risk of infection and challenges in investigation among marginalized workers without comprehensive benefits or stable working conditions. The investigation also adds to the evidence for expanding the analysis window for *S.* Typhi WGS cluster assignment.
